# Metabolomic profiling in a Hedgehog Interacting Protein (Hhip) murine model of chronic obstructive pulmonary disease

**DOI:** 10.1038/s41598-017-02701-4

**Published:** 2017-05-31

**Authors:** Emily S. Wan, Yan Li, Taotao Lao, Weiliang Qiu, Zhiqiang Jiang, John D. Mancini, Caroline A. Owen, Clary Clish, Dawn L. DeMeo, Edwin K. Silverman, Xiaobo Zhou

**Affiliations:** 1 Channing Division of Network Medicine, Brigham and Women’s Hospital, Boston, MA USA; 2 Division of Pulmonary and Critical Care, Brigham and Women’s Hospital, Boston, MA USA; 3 Pulmonary Section, VA Boston Healthcare System, Jamaica Plain, MA USA; 4 Lovelace Respiratory Research Institute, Albuquerque, NM USA; 5 Broad Institute, Cambridge, MA USA

## Abstract

Genetic variants annotated to the hedgehog interacting protein (*HHIP*) are robustly associated with chronic obstructive pulmonary disease (COPD). *Hhip* haploinsufficiency in mice leads to increased susceptibility towards the development of emphysema following exposure to chronic cigarette smoke (CS). To explore the molecular pathways which contribute to increased susceptibility, we performed metabolomic profiling using high performance liquid chromatography tandem mass spectroscopy (LC/MS-MS) on plasma, urine, and lung tissue of *Hhip* ^+/−^ heterozygotes and wild type (*Hhip* ^+/+^) C57/BL6 mice exposed to either room-air or CS for six months. Univariate comparisons between groups were made with a combined fold change ≥2 and Student’s t-test p-value < 0.05 to denote significance; associations with mean alveolar chord length (MACL), a quantitative measure of emphysema, and gene-by-environment interactions were examined using empiric Bayes-mediated linear models. Decreased urinary excretion of cotinine *despite* comparable plasma levels was observed in *Hhip* ^+/−^ heterozygotes; a strong gene-by-smoking association was also observed. Correlations between MACL and markers of oxidative stress such as urinary methionine sulfoxide were observed in *Hhip* ^+/−^ but not in *Hhip* ^+/+^ mice. Metabolite set enrichment analyses suggest reduced antioxidant capacity and alterations in macronutrient metabolism contribute to increased susceptibility to chronic CS-induced oxidative stress in *Hhip* haploinsufficiency states.

## Introduction

Chronic obstructive pulmonary disease (COPD), the third leading cause of death in the United States^1^, is a complex disease influenced by both genetic and environmental risk factors. Recent genome-wide association studies (GWAS) have consistently identified a COPD susceptibility locus in an intergenic region on chromosome 4q31^2^. Using chromosomal conformation capture studies, our group demonstrated that this GWAS region interacts with the hedgehog interacting protein (*HHIP*) promoter through a functional genetic variant within a distal enhancer which alters binding to the SP3 transcription factor^3^. Subsequent studies of human lung epithelial cells exposed to cigarette smoke *in vitro* suggested that, in addition to its established roles in morphogenesis and embryonic development through the hedgehog pathway^4^, *HHIP* may alter extracellular matrix and cell growth pathways^5^. Recently, we also demonstrated a role for HHIP in the development of spontaneous, age-related emphysema in murine models of *Hhip* haploinsufficiency^6^.

To explore the interaction between *HHIP* and exposure to chronic cigarette smoke (CS), the leading environmental risk factor for COPD, we studied a murine model of *HHIP* haploinsufficiency generated on a C57/BL6 background. While homozygous *Hhip* ^−/−^ mice die shortly after birth due to defects in lung branching morphogenesis, *Hhip* ^+/−^ heterozygotes are viable, have normal lung development, and exhibit an approximately 33% reduction in the expression of *Hhip*, a level comparable to that observed in human COPD lung tissue samples harboring *HHIP* GWAS risk variants^3^. *Hhip* ^+/−^ heterozygote mice demonstrate an increased susceptibility towards the development of both functional and histological emphysema when exposed to chronic cigarette smoke^3, 7^; network analysis of lung gene expression data demonstrated an enrichment of lymphocyte activation pathways in *Hhip* ^+/−^ mice relative to similarly exposed wild type mice^7^. To date, investigations into the metabolic perturbations which may contribute to the increased susceptibility towards the development of emphysema in *Hhip* ^+/−^ heterozygotes have not been performed.

Metabolomic profiling is a relatively novel “-omics” platform where the comprehensive small molecule composition of a biological material is assessed. As such, metabolomics represents a more proximal and integrative snapshot of the environmental and genetic risk factors which likely contribute to a disease phenotype. Unlike the genetic profile which remains largely invariant within a given individual, multiple “metabolomes” representing different biological materials, physiological states, and exposure conditions can exist in a single organism. To further explore the impact of *HHIP* haploinsufficiency on the development of COPD, metabolomic profiling using an untargeted liquid chromatography-tandem mass spectroscopy (LC-MS/MS) platform was performed on the plasma, urine, and lung tissue from *Hhip* ^+/−^ heterozygote and *Hhip* ^+/+^ wild type mice exposed to either room air or 6 months of cigarette smoke (2 × 2 experimental design).

## Results

Five mice were analyzed from each group in this 2 × 2 experimental design: room air-exposed *Hhip* ^+/+^, CS-exposed *Hhip* ^+/+^, room air-exposed *Hhip* ^+/−^, and CS-exposed *Hhip* ^+/−^. Although profiling was performed using an untargeted platform, because we wished to perform downstream pathway and enrichment analyses, we limited our analyses to identified compounds. In total, 319 compounds were identified in plasma, 197 in urine, and 323 in lung. Fatty acids were not detected in urine samples and account for the decreased number of metabolites reported in urine. The overlap between metabolites in each dataset by sample type are shown qualitatively in Supplementary Figure S1.

### Baseline differences in metabolites by Hhip genotype

The spectral peak intensity of each metabolite, which is proportional to concentration, was analyzed following log transformation and Pareto scaling. Metabolites demonstrating a minimum ≥2 fold change and a Students t-test p-value (unadjusted) <0.05 between groups were considered significant. Differences in metabolism by *Hhip* genotype (*Hhip* ^+/−^ heterozygotes versus *Hhip* ^+/+^ wild type) under room air conditions are shown in Table 1. Both C6 and C8 carnitine are significantly reduced in plasma samples from *Hhip* ^+/−^ mice relative to *Hhip* ^+/+^ mice. No metabolite met both the fold change (≥2) and statistical threshold for significance in lung tissue.

**Table 1:** Metabolites with differential concentrations by Hhip genotype (Hhip^+/−^ versus Hhip^+/+^) in room air-exposed mice.

Sample Type/Metabolite	Log2 (Fold change)*	P-value†
*Plasma*		
C6 carnitine	−2.58	0.02
C8 carnitine	−1.41	0.02
*Urine*		
2-deoxyadenosine	−1.17	0.04
Adenine	1.5	0.01
Histidine	1.14	0.01
Pyroglutamic acid	−1.3	0.01

*Negative values indicate *lower* concentration in Hhip^+/−^ heterozygotes (minimum 2x fold change).

^†^Student’s t-test. No metabolites met the thresholds for significance in lung tissue.

### Impact of chronic cigarette smoke exposure on metabolite levels

**Figure 1:**
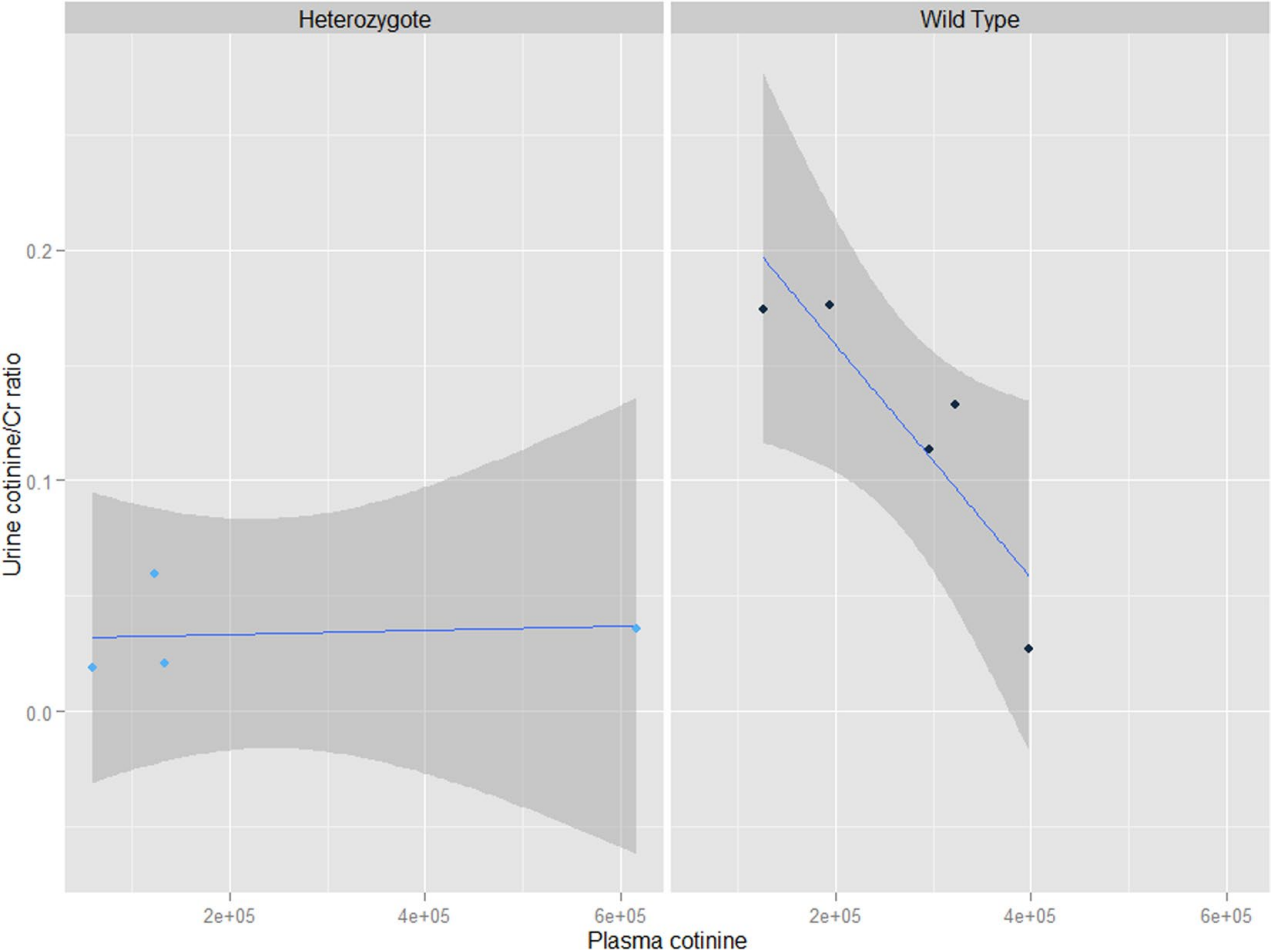
Urinary and plasma cotinine levels by genotype. Urinary cotinine (normalized for creatinine concentration) relative to plasma cotinine in *Hhip* ^+/−^ heterozygotes (left panel) and *Hhip* ^+/+^ wild type mice (right panel) subjected to chronic cigarette smoke. In *Hhip* ^+/+^ wild type mice, a strong inverse correlation between urine and plasma cotinine levels exist (Pearson rho = −0.89, p-value = 0.04) whereas in *Hhip* ^+/−^ heterozygotes, no correlation was found (Pearson rho = 0.12, p-value = 0.88). The best fit line is plotted in blue while the 95% confidence interval is plotted in dark gray.

Exposure to cigarette smoke increased the number of metabolites which exist at different concentrations relative to baseline (room-air) conditions in both *Hhip* ^+/+^ (Table 2) and *Hhip* ^+/−^ mice (Table 3). As a proof of concept, increased levels of cotinine, an established metabolite of nicotine and biomarker of cigarette smoke exposure, were consistently identified in both the plasma and urine of CS-exposed mice relative to room air-exposed mice. Interestingly, while a strong inverse correlation between urinary and plasma cotinine levels was noted among *Hhip* ^+/+^ wild type mice exposed to chronic CS, this relationship was *not* observed in *Hhip* ^+/−^ heterozygotes (Fig. 1) and may suggest genotype-dependent differences in cotinine metabolism.

**Table 2:** Metabolites with differential concentrations following exposure to chronic cigarette smoke exposure in wild-type (Hhip^+/+^) mice.

Sample Type/Metabolite	Log2 (Fold change)*	P-value†
*Plasma*		
Cotinine	5.2	2.48 × 10^−3^
Glutathione (oxidized)	−1.01	0.03
*Urine*		
1-methylhistamine	−1.07	2.91 × 10^−3^
5-aminolevulinic acid	2.42	0.02
Adenine	1.46	7.34 × 10^−3^
Creatine	−2.54	7.52 × 10^−4‡^
Cotinine	4.61	7.69 × 10^−4‡^
Guanine	1.53	2.38 × 10^−6‡^
N-carbamoyl-beta-alanine	−1.04	0.02
Nicotinate	4.36	0.02
Oxalate	1.17	0.05
Pantothenate	−1.78	0.04
Xanthine	1.34	6.16 × 10^−3^

*Negative values indicate *lower* concentration in wild type Hhip^+/+^ mice exposed to chronic cigarette smoke (minimum 2x fold change).

^†^Student’s t-test p-value.

^‡^Denotes significance at a false discovery rate (FDR) < 0.05.

No metabolites met the thresholds for significance in lung tissue.

**Table 3:** Metabolites with differential concentrations following exposure to chronic cigarette smoke exposure in (Hhip^+/−^) heterozygote mice.

Sample Type/Metabolite	Log2 (Fold change)*	P-value^†^
*Plasma*		
4-hydroxybenzaldehyde	1.57	0.03
AMP	2.33	0.02
Cotinine	3.95	1.55 × 10^−3^
Cytidine	1.39	0.02
Gentisate	−1.44	0.01
Glucose	−1.06	0.04
Glutamate	1.29	0.01
GMP	1.85	0.02
Threonine	1.11	0.04
UMP	2.53	0.03
*Urine*		
ADP	1.13	0.03
Alpha-ketoglutarate	−1.55	0.02
AMP	1.52	0.02
Argininosuccinate	−1.72	1.05 × 10^–3^
C3-DC-CH3 carnitine	1.38	0.04
Carnosine	−1.73	0.04
Cotinine	2.56	0.02
Glutamate	1.02	0.03
Guanine	1.13	9.67 × 10^−3^
Histidine	−1.34	3.95 × 10^−3^
Lactate	−1.22	0.01
Malate	−1.24	0.03
Pantothenate	−1.41	0.02
Putrescine	−1.75	0.03
Succinate	−1.05	5.83 × 10^−3^
XMP	1.91	0.02
*Lung*		
Adenylosuccinate	1.41	4.98 × 10^−3^

*Negative values indicate *lower* concentration in Hhip^+/−^ mice exposed to chronic cigarette smoke (minimum 2x fold change).

^†^Student’s t-test.

When we compared *Hhip* ^+/+^ wild type to *Hhip* ^+/−^ heterozygotes exposed chronic CS, a more modest number of metabolites were identified as existing in different concentrations by genotype (Table 4). Significantly lower concentrations of cotinine were observed in the urine of *Hhip* ^+/−^ relative to *Hhip* ^+/+^ mice. Again, no metabolites met both the ≥2 fold change and statistical threshold for significance in lung tissue.

**Table 4:** Metabolites with differential concentrations by Hhip genotype (Hhip^+/−^ versus Hhip^+/+^) in cigarette smoke-exposed mice.

Sample Type/Metabolite	Log2 (Fold change)*	P-value†
*Plasma*		
C30:1 phosphatidylcholine	−1.51	0.04
Pantothenate	1.16	3.6 × 10^−3^
Sorbitol	1.39	0.05
*Urine*		
Alpha-hydroxybutyrate	−1.22	0.04
C5 carnitine	−1.82	0.03
Cotinine	−1.89	0.03
Creatine	1.17	0.03

*Negative values indicate *lower* concentration in Hhip^+/−^ heterozygotes (minimum 2x fold change).

†Student’s t-test.

No metabolites met the thresholds for significance in lung tissue.

Because of the relative paucity of metabolites which met our stringent criteria for significance in lung tissue in the majority of our analyses, we performed secondary exploratory analyses using a less stringent threshold of 1.5x fold change (while keeping the statistical threshold of p < 0.05 unchanged) to denote significance. Comparisons between *Hhip* ^+/+^ and *Hhip* ^+/−^ mice exposed to room air (Supplementary Table S1), differences by smoke exposure in *Hhip* ^+/+^ (Supplementary Table S2) and *Hhip* ^+/−^ (Supplementary Table S3) mice, and differences by genotype when both groups were exposed to chronic CS (Supplementary Table S4) were examined. Wild-type *Hhip* ^+/+^ demonstrate increases in 2 phosphatidylcholine compounds (C30:1 PC and C32:2 PC) in lung upon exposure to chronic CS; these increases are not observed in *Hhip* ^+/−^ heterozygotes; the differences in C30:1 PC and C32:2 PC are significant when comparing *Hhip* ^+/+^ and *Hhip* ^+/−^ groups that have both been exposed to chronic CS. Additionally, reduced levels of gentisate were observed in the plasma and lung tissue of *Hhip* ^+/−^ exposed to chronic CS.

### Gene-by-smoking analyses

To explore gene-by-environment interactions, we constructed empiric Bayes’ mediated linear models with raw metabolite peak intensities as the dependent variable; dichotomized variables for the main effects and a multiplicative interaction term were modeled as the independent variables as shown below:

$$[{\rm{Metabolitepeakintensity}}] \sim [{\rm{Genotype}}]+[{\rm{Smoking}}]+[\mathrm{Genotype}:\mathrm{Smoking}]$$

Metabolites with an association p-value < 0.05 for the interaction term were considered significant. Metabolites with significant gene-by-environment interactions are listed in Table 5. Four of the six metabolites identified in plasma were also identified in urine; the two non-overlapping metabolites in plasma are lipid derivatives which would not be expected to be present in urine. Using permutation testing to determine the null distribution for the expected overlap between plasma and urine metabolites, an overlap of ≥4 metabolites is significantly greater than would be expected by chance (p_permutation_ = 0.002). Only fructose/glucose/galactose were identified in both the urine and lung analyses while C58:12 triacylglycerol overlapped between the plasma and lung analyses.

**Table 5:** Metabolites with significant (p < 0.05) gene-by-environment interactions.

Sample Type/Metabolite	
*Plasma*	
Aconitate	
Adipate	
C22:6 Lysophosphatidylcholine	
C58:12 Triacylglycerol	
Isocitrate	
Thymine	
*Urine*	
2-deoxyadenosine	Fructose/glucose/galactose
2-hydroxyglutarate	Hydroxyphenylpyruvate
3-hydroxybenzoate	Inositol
3-methyladipate	Isocitrate
4-hydroxybenzaldehyde	Isoleucine^‡^
5-adenosylhomocysteine	N-carbamoyl-beta-alanine
Aconitate^‡^	Phosphocholine
Adenosine	Pyroglutamic acid
Adipate	Salicylurate
Alpha-glycerophosphate	Symmetric dimethylarginine
Alpha-glycerophosphocholine	Suberate
Asparagine	Taurocholate^‡^
Asymmetric dimethylarginine	Thymine
cAMP	Tryptophan
chenodeoxycholate	Tyrosine
creatinine	Uracil
cotinine	Valine
cytosine	Xanthosine
*Lung*	
2-hydroxyglutarate	
2-phosphoglycerate	
3-phosphoglycerate	
Alpha-hydroxybutyrate	
Betaine	
Butyrobetaine	
C14:0 sphingomyelin	
C22:0 sphingomyelin	
C32:2 phosphatidylcholine	
C58:12 triacylglycerol	
Fructose/glucose/galactose	
Lactose	
Malondialdehyde	
Sucrose	

^‡^Denotes significance at a false discovery rate (FDR) < 0.05.

### Association with histological emphysema severity

The mean alveolar chord length (MACL), a quantitative histological measurement of the average vertical and horizontal distances between the alveolar walls, was used as a surrogate for emphysema severity and was determined according to previously published protocols^8^. Empiric Bayes-mediated linear models were constructed for each experimental condition using the raw metabolite peak values and mean MACL as the dependent and independent variables, respectively; metabolites with an association p-value < 0.05 were considered significant.

**Figure 2:**
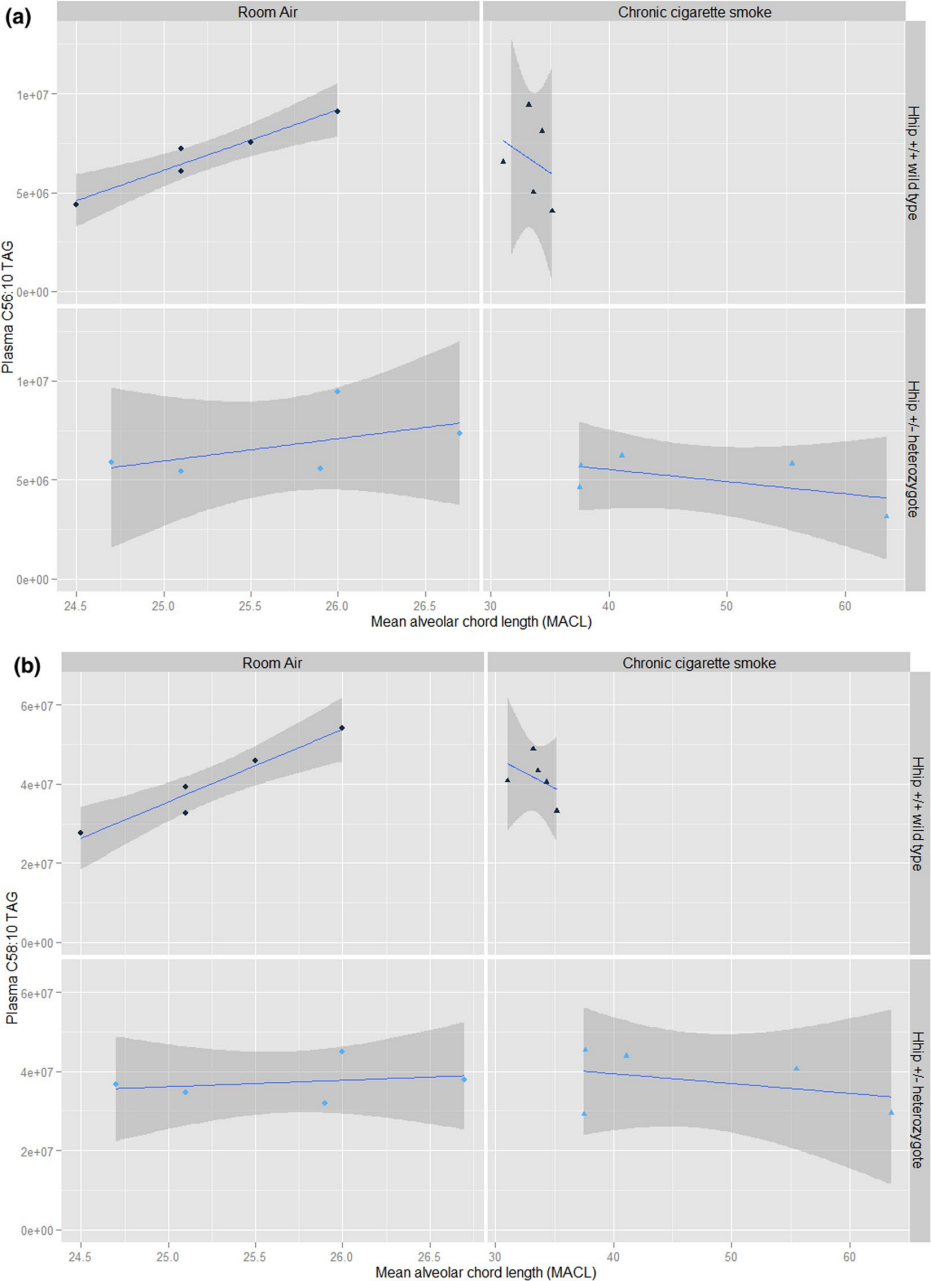
Association between plasma C56:10 (panel A) and C58:10 (panel B) triacylglycerol (TAG) and lung mean alveolar chord length (MACL) by experimental condition. A strong correlation between C56:10 TAG (Panel A, Pearson’s rho = 0.97, p-value = 7.22 × 10^–3^) and C58:10 TAG (Panel B, Pearson’s rho = 0.97, p-value = 7.14 × 10^–3^) and MACL is observed *Hhip* ^+/+^ wild type exposed to room air which is not observed in *Hhip* ^+/+^ wild type exposed to chronic cigarette smoke or in *Hhip* ^+/−^ heterozygotes exposed to either experimental condition. The best fit line is plotted in blue while the 95% confidence interval is plotted in dark gray.

**Figure 3:**
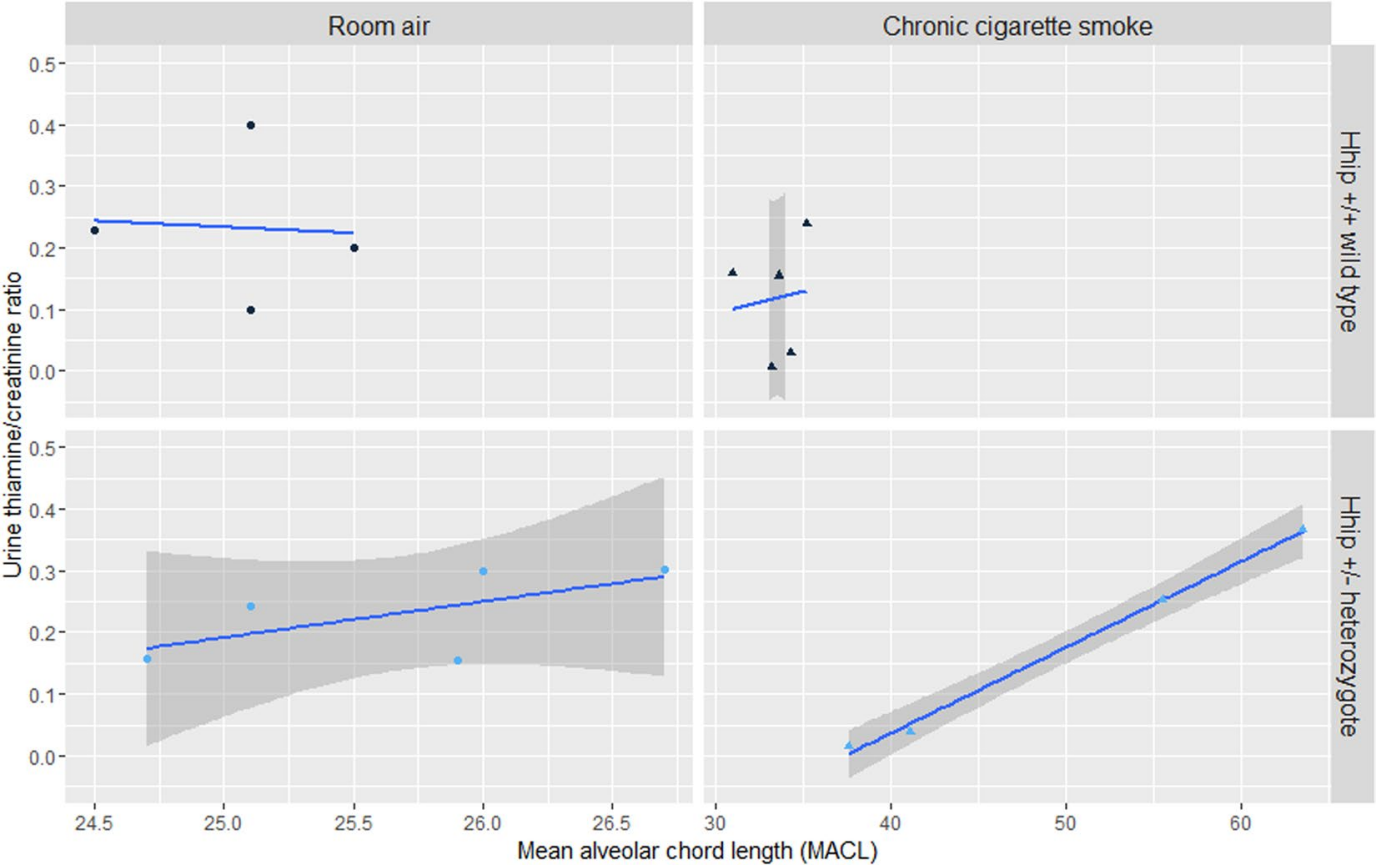
Association between urinary thiamine/creatinine ratio and lung mean alveolar chord length (MACL). A significant association between urinary thiamine/creatinine was noted in *Hhip* ^+/−^ heterozygotes exposed to chronic cigarette smoke (CS) (lower right panel, Pearson’s rho = 0.99, p-value = 1.67 × 10^–3^). No association was observed in *Hhip* ^+/−^ mice exposed to room air or in *Hhip* ^+/+^ wild type mice in either experimental condition. The best fit line is plotted in blue while the 95% confidence interval is plotted in dark gray (except *Hhip* ^+/+^, room air, where wide 95%CI exceeds panel borders).

**Figure 4:**
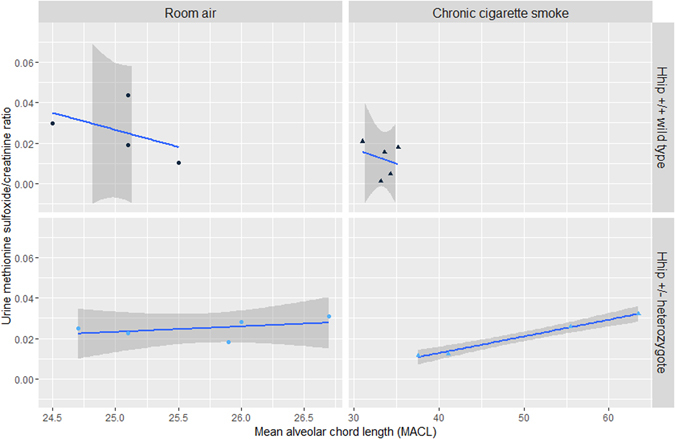
Association between urinary methionine sulfoxide/creatinine ratio and lung mean alveolar chord length (MACL). A significant association between urinary methionine sulfoxide/creatinine was noted in *Hhip* ^+/−^ heterozygotes exposed to chronic cigarette smoke (CS) (lower right panel, Pearson’s rho = 0.99, p-value = 3.94 × 10^–3^). No association was observed in *Hhip* ^+/−^ mice exposed to room air or in *Hhip* ^+/+^ wild type mice in either experimental condition. The best fit line is plotted in blue while the 95% confidence interval is plotted in dark gray.

Results are shown in Supplementary Tables S5 (plasma), S6 (urine) and S7 (lung). A strong association between two lipids, C56:10 and C58:10 triacylglycerol (TAG), and MACL was noted in the plasma of *Hhip* ^+/+^ mice exposed to room air; these associations were no longer present following exposure to chronic CS nor was it present in *Hhip* ^+/−^ heterozygotes under either experimental condition (Fig. 2, panels A and B, respectively). Urinary thiamine and methionine sulfoxide levels (normalized to creatinine) were significantly associated with MACL in *Hhip* ^+/−^ heterozygotes exposed to chronic CS (Figs 3 and 4, respectively). No correlation was observed in *Hhip* ^+/−^ mice exposed to room air or in *Hhip* ^+/+^ wild type mice exposed to either experimental condition.

### Metabolite set enrichment analysis (MSEA) and pathway analyses

Metabolites significant in the (a) univariate analyses by both fold change and p-value testing, (b) gene-by-smoking analysis, and (c) MACL analyses above were mapped to the appropriate unique identifier in the Human Metabolome Database (HMDB ID); these identifiers were used as input for MSEA and pathway analyses as implemented in MetaboAnalyst 3.0^9^; a false discovery rate (FDR) ≤ 0.05 was considered significant.

**Figure 5:**
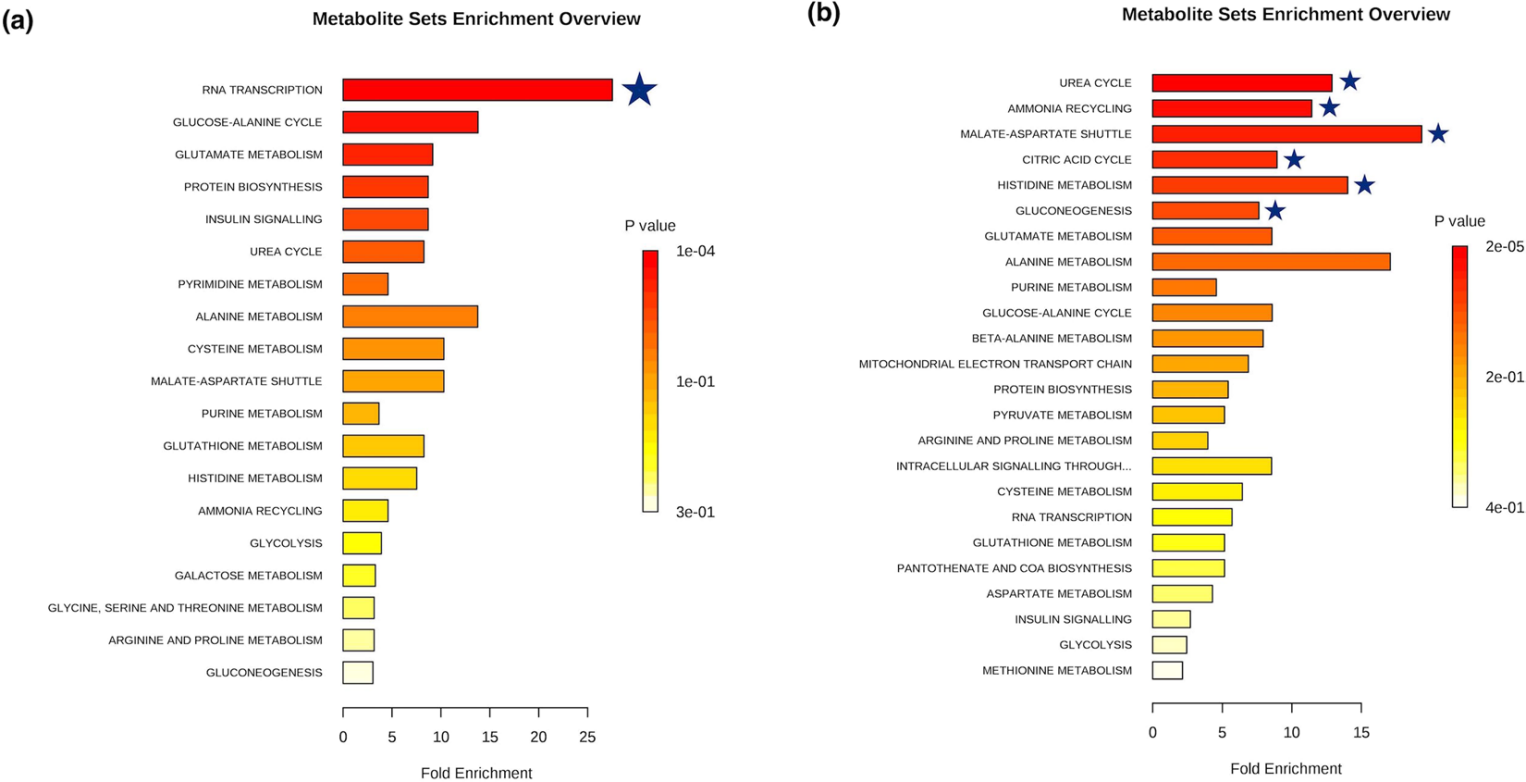
Metabolite set enrichment analysis based on differentially expressed metabolites identified in plasma (**a**) and urine (**b**) from *Hhip* ^+/−^ heterozygotes exposed to chronic cigarette smoke relative to mice exposed to room air. Metabolite sets significant at an FDR < 0.05 are denoted with a blue star.

Of the metabolite groups identified in the univariate comparisons, metabolites from the analysis of *Hhip* ^+/−^ heterozygotes mice exposed to chronic CS versus room air were enriched for several processes. An enrichment of plasma metabolites annotated to RNA transcription was observed, while metabolites annotated to the urea cycle, ammonia recycling, and the citric acid cycle were enriched in the urine (Fig. 5, panels a and b, respectively). Metabolite groups from other univariate comparisons did not demonstrate significant enrichments on either MSEA or pathway analyses.

When we performed a similar analysis on metabolites identified in the gene-by-smoking analysis, a trend towards enrichment in metabolites annotated to protein synthesis (FDR = 0.07) was identified in the analysis of urine. As a subgroup analysis, we performed pathway enrichment analysis using the 4 overlapping metabolites identified in both plasma and urine gene-by-environment analyses and noted a significant enrichment of metabolites annotated to (1) glyoxylate and dicarboxylate metabolism and (2) the citrate cycle pathways (Supplementary Figure S2). Pathway analysis of metabolite groups associated with MACL in the urine of Hhip^+/+^ exposed to room air demonstrated a trend towards enrichment of metabolites annotated to glycerophospholipid metabolism (FDR = 0.054).

### Validation of selected metabolites

To confirm the results of our mass spectroscopy-based metabolomic profiling data, we performed ELISA-based assays for cotinine and creatine on samples collected from a largely non-overlapping cohort of mice from the same experimental conditions. Results of the quantitative urinary cotinine assay are shown in Supplementary Figure S3. Significantly higher amounts of cotinine were detected in the urine of both *Hhip* ^+/+^ and *Hhip* ^+/−^ mice exposed to chronic CS relative to those exposed to room air; these findings are consistent with the mass spectroscopy-based metabolomics data. However, no significant difference in urinary cotinine was noted between *Hhip* ^+/+^ and *Hhip* ^+/−^ when both were exposed to chronic CS; this may be due to limited sample size or differential sensitivity between the two assays. Serum cotinine levels are illustrated in Supplementary Figure S4. Surprisingly, while there was a trend towards higher cotinine levels in both *Hhip* ^+/+^ and *Hhip* ^+/−^ mice exposed to chronic CS relative to mice exposed to room air, the differences did *not* meet the thresholds for statistical significance in either group. We also validated the effects of chronic CS on creatine, an organic acid involved in energy supply which is largely stored in skeletal muscle cells. Decreased levels of urinary creatine were observed in *Hhip* ^+/+^, but *not* in *Hhip* ^+/−^, mice after chronic CS exposure (Supplementary Figure S5). There were, however, no differences in urinary creatine levels by genotype when we compared *Hhip* ^+/+^ to *Hhip* ^+/−^ mice after both were exposed to chronic CS. While most of samples assayed in the validation cohort were from an *independent group* of mice, one mouse overlapped with the original cohort profiled using the untargeted mass-spectroscopy based metabolomic profiling. Exclusion of the single overlapping mouse did not change the significance of the results reported in each of the comparisons above.

## Discussion

The hedgehog family of proteins is classified as growth factors and morphogens which mediate an expansive number of processes during embryogenesis and development^10^. Hedgehog interacting protein (*HHIP*) is a highly conserved, vertebrate-specific protein which is both induced by and serves as a negative regulator of hedgehog signaling^11^. *HHIP* has an established role in branching morphogenesis of the lung during embryonic development^4^; based on subsequent RNA interference studies in a human airway epithelial cell line, *HHIP* was also implicated in lung extracellular matrix and cell growth pathways^5^. In addition to associations with adult height^12^ and several malignancies^13^, variants annotated to the *HHIP* locus have been robustly associated with lung function and the development of COPD^2, 14^. To date, the mechanisms which underlie the association between *HHIP* and late-onset (adult) complex diseases have not been fully characterized. A murine model of *Hhip* haploinsufficiency which closely mimics the impact of genetic variants identified by genome-wide association studies on the gene expression of *HHIP* in the lung demonstrates increased susceptibility towards the development of emphysema upon exposure to chronic cigarette smoke^7^; using this model, we explored both baseline differences in metabolism as well as differences induced by exposure to a significant environmental challenge (i.e., chronic cigarette smoke exposure).

While complete loss of *Hhip* function results in perinatal lethality, haploinsufficiency at the *Hhip* locus does not appear to significantly alter the viability or lung morphology relative to wild-type mice^7^ under normal conditions. Likewise, baseline differences in metabolism between *Hhip* ^+/−^ and *Hhip* ^+/+^ mice also appear to be modest. Decreased levels of C6 and C8 carnitine were detected in the plasma of *Hhip* ^+/−^ heterozygotes; both compounds belong to the broader category of acyl carnitines, a group of metabolites involved in fatty acid oxidation. Interestingly, *increased* plasma levels of C6 and C8 carnitine are characteristic of medium chain acyl-CoA dehydrogenase deficiency (MCAD; OMIM 201450), an autosomal recessive disorder characterized by an intolerance to fasting^15, 16^. Although the functional impact of *decreased* levels of plasma C6 and C8 carnitine is not known, decreased levels of L-carnitine in lung tissues were recently reported in a porcine pancreatic elastase murine model of emphysema^17^.

Exposure to cigarette smoke appears to elicit a number of changes to metabolite concentrations in the plasma and urine of both *Hhip* ^+/+^ wild type and *Hhip* ^+/−^ heterozygotes. Decreased urinary excretion of pantothenate, an essential nutrient also known as vitamin B5 which is involved in the synthesis of coenzyme A (CoA) and the metabolism of almost all macronutrients (proteins, carbohydrates, and fats), was observed in both *Hhip* ^+/+^ and *Hhip* ^+/−^ mice exposed to cigarette smoke. In both instances, a corresponding increase in plasma levels was *not* observed. Thus, whether cigarette smoke exposure alters the synthesis or absorption of the nutrient from the gastrointestinal tract or the localization, turnover, or sequestration of pantothenate in different tissues remains unknown. Interestingly, increased levels of pantothenate have been reported in a human alveolar lung tissue cell line exposed to mainstream cigarette smoke *in vitro* ^18^; this was not observed in our data which was ascertained on whole lung tissue samples from an *in vivo* exposure model.

Another metabolite consistently altered in both *Hhip* ^+/+^ and *Hhip* ^+/−^ mice exposed to chronic CS was guanine, a purine derivative which is one of five bases integral to DNA and RNA. Increased urinary guanine in CS-exposed mice was one of the strongest associations in our study, remaining robust even after application of a conservative Bonferroni adjustment for multiple testing. Interestingly, several derivatives of guanine, such as 8-hydroxydeoxyguanosine and 7-methylguanine, have been associated with cigarette smoking and may reflect levels of oxidative damage and DNA methylation, respectively^19–21^; these guanine derivatives were not assessed directly in our study and represent potential avenues for future investigations.

Cotinine, a major pharmacologically-active metabolite of nicotine, is an established biomarker of both active and passive cigarette smoke exposure^22^ and was significantly increased in the urine and plasma of CS-exposed *Hhip* ^+/+^ and *Hhip* ^+/−^ mice. The finding of increased levels of cotinine and a relative depletion of glutathione in the plasma of *Hhip* ^+/+^ wild type mice exposed to chronic cigarette smoke is also consistent with known hepatic xenobiotic detoxification pathways^23^ and serves as a proof-of-concept finding. Interestingly, when we compared *Hhip* ^+/+^ and *Hhip* ^+/−^ mice which had both been exposed to chronic CS, *Hhip* ^+/−^ heterozygotes had significantly lower urinary cotinine relative to *Hhip* ^+/+^ wild type mice. The decreased urinary excretion of cotinine in *Hhip* ^+/−^ heterozygotes occurs despite similar plasma cotinine levels in both strains and does not appear to be due to differential renal function as assessed by plasma creatinine levels (p-value = 0.86). Previous studies have demonstrated a strong correlation between plasma and urinary levels of cotinine in both humans and mice ^22, 23^; a strong inverse correlation between plasma and urinary cotinine levels was noted for *Hhip* ^+/+^ wild type mice exposed to cigarette smoke (Pearson’s rho = −0.89, p-value = 0.04), but no correlation was noted in *Hhip* ^+/−^ heterozygotes (Pearson’s rho = 0.12, p-value = 0.88) (Fig. 1). These results are consistent with the finding of a strong gene-by-smoking effect for cotinine identified in our earlier analysis (Table 5). Whether cotinine is differentially metabolized into alternative or downstream metabolites, such as trans-3-hydroxy-cotinine or cotinine-N-oxide by *Hhip* ^+/−^ heterozygotes remains unknown as the majority of nicotine and cotinine derivatives remain unannotated in our dataset.

We were able to support some of the mass spectroscopy-based results reported above using ELISA and colorimetric assays in a largely non-overlapping cohort of mice from the same experimental conditions. Urinary cotinine excretion was increased among both *Hhip* ^+/+^ and *Hhip* ^+/−^ mice exposed to chronic CS relative to mice exposed to room air, however no difference in cotinine excretion was found when we compared *Hhip* ^+/+^ and *Hhip* ^+/−^ mice who had *both* been exposed to chronic CS. However, due to limited sample availability, urinary creatinine levels could not be assayed, thus these values were not normalized to urinary creatinine excretion. Interestingly, while a trend towards increased plasma cotinine was observed in both *Hhip* ^+/+^ and *Hhip* ^+/−^ mice exposed to chronic CS, these differences did not reach statistical significance in either group despite *known and controlled exposure* to cigarette smoke. Whether this is due to the rapid clearance of cigarette smoke metabolites in plasma or due to limitations of the assay employed remains undetermined. We similarly support the mass spectroscopy-based findings of reduced in urinary creatine observed in *Hhip* ^+/+^ mice exposed to chronic CS as well as the *lack* of decrease in urinary creatine excretion in *Hhip* ^+/−^ heterozygotes. There was, again, no statistically significant difference by genotype when comparing the two groups of mice exposed to chronic CS, but these values were similarly not adjusted for urinary creatinine excretion.

The mechanisms which contribute to the propensity of *Hhip* ^+/−^ heterozygotes towards developing more severe histological and functional emphysema when exposed to chronic CS or during aging relative to their wild type counterparts are incompletely understood but are likely mediated through an increased sensitivity towards oxidative stress^6^. Given the “standardized” exposure to oxidative stress in our experiment, we hypothesize that this increased sensitivity may be due to a) reduced antioxidant capacity and b) changes in metabolism which perpetuate oxidative damage. Findings which support the hypothesis of reduced antioxidant capacity in *Hhip* ^+/−^ heterozygotes include reduced levels of gentisate in plasma and lung (Table 3 and Supplementary Table S3) following exposure to CS and the strong association between histological emphysema and urinary excretion of methionine sulfoxide, a biological marker of oxidative stress and aging. Gentisate, an intermediate in both salicylic acid and benzoate metabolism, has been shown to have antioxidant and free-radical scavenging properties *in vitro* ^24^; depletion in plasma and lung is thus biologically plausible. Methionine residues in protein complexes also demonstrate similar scavenging properties and play a significant role as endogenous antioxidants; exposure to reactive oxygen species leads to the formation of methionine sulfoxide and reflects the oxidative burden experienced by the organism^25^. Regeneration of methionine through methionine sulfoxide reductases allows the cell/organism to retain the protein’s structural integrity as well antioxidant capacity. Whether *Hhip* directly impacts methionine sulfoxide reductase function is a potentially intriguing area for future studies.

Changes in metabolism in *Hhip* ^+/−^ heterozygotes which may *perpetuate* oxidative damage are based upon evidence of differential macronutrient utilization which supports increased dependence on glucose and carbohydrate metabolism and reduced reliance upon fatty acid metabolism. In metabolite set enrichment analysis, the citric acid cycle, malate-aspartate shuttle, and gluconeogenesis were significantly enriched (Fig. 5). Thiamine, which was strongly correlated with histological emphysema in *Hhip* ^+/−^ heterozygotes (Fig. 3), is also an essential micronutrient integral to glucose metabolism^26^. This increased reliance upon carbohydrate metabolism, which has been shown to cause increased oxidative stress relative to fatty acid metabolism^27^, has similarly been demonstrated in human COPD subjects^28, 29^.

The net result of increased oxidative burden from both of the mechanisms above is disrupted proteostasis, with increased degradation of structural proteins due to damage and shunting of amino acids into pathways to serve as substrates for the citric acid cycle to meet essential energy requirements. Increased protein turnover and degradation is supported by the finding of enrichment for metabolites annotated to ammonia recycling, the urea cycle, and histidine metabolism in *Hhip* ^+/−^ mice (Fig. 5), with similar disruptions in metabolism described in human COPD patients^30^. Examining specific disrupted pathways and potential therapeutic strategies represent future directions of investigation.

A unique strength of our data involves the simultaneous profiling of several sample types at once, an approach which allowed us to leverage established physiology and biochemistry in the interpretation of our findings. In addition to expanding our insight into the specific metabolic pathways which may be involved in *HHIP*-mediated susceptibility towards developing COPD, we made several general observations. First, we noted a relative lack of significant metabolites on univariate analyses of lung tissue despite the fact that this tissue was directly exposed to cigarette smoke. Previous studies utilizing alternative pulmonary-derived samples, such as expectorated sputum, bronchoalveolar lavage fluid, or exhaled breath condensate, have reported differences in metabolism between smokers, non-smokers, and COPD subjects^31, 32^. We speculate that the relative paucity of significant findings in lung tissue may be due to tissue and cell-type heterogeneity as profiling was performed on lung homogenates as opposed to largely acellular biofluids such as plasma and urine. We additionally addressed this in our exploratory secondary analyses using a less stringent fold-change threshold for all metabolites and identify additional potentially interesting metabolites altered by either genotype or cigarette smoke exposure. A second observation was that in both the univariate and gene-by-environment analyses, the number of significantly associated metabolites was consistently highest in urine. Increased sensitivity to differences in metabolite concentrations in urine relative to plasma has been previously reported for tobacco-related metabolites^33, 34^; this was validated in our ELISA cotinine assay which found greater sensitivity in detecting differences by known exposure status in urine relative to plasma. Our metabolomic profiling analyses suggest that, in addition to increased sensitivity, urinary metabolomic profiling may offer *complementary* information on metabolic processes relative to plasma metabolomic profiling alone. Given the abundance and relative ease of collection, as well as expanding resources cataloging compounds and reference values^35^, urinary metabolomic profiling may be a promising avenue for large-scale metabolomic investigations in the future.

Our exploratory analyses using metabolomic profiling of a murine model of *HHIP* haploinsufficiency have generated a hypothesis that *HHIP* could play a role in the development of COPD through differential handling of environmental toxins and increased sensitivity towards oxidative damage; further studies will be required to assess this hypothesis. We assert that contemporaneous profiling of plasma and urine offers complementary information on metabolic processes affected by environmental exposures. We acknowledge limited power to detect small to moderate differences in metabolites due to the small number of animals in each experimental condition but contend that the use of genetically identical mice and a highly controlled exposure model, as well as validation of selected metabolites using independent technologies, contribute to the rigor of the experiment and the reported findings. We acknowledge that the majority of metabolites identified in our analyses would not remain significant after correction for multiple testing and that a certain proportion of our associations may represent false positive findings. However, we assert that the inclusion of a fold-change threshold, as well as the identification of biologically plausible changes in metabolites supports the validity our findings. Regarding our gene-by-environment analysis, we acknowledge our use of a multiplicative interaction term does not assess for other possible modes of interaction (i.e., additive effects); future studies involving a larger number of observations will allow for more detailed analyses of more complex interactions. Lastly, we acknowledge that, because our model is murine and examined only female mice, the generalizability of our findings to both genders and extrapolation to human metabolism is not known; thus, future metabolomic investigations in both genders and using human populations are warranted.

## Materials and Methods

### Murine model and sample collection

Details regarding the generation of *Hhip* ^+/−^ mice and the chronic cigarette smoke exposure conditions have been previously published^7^. All protocols were in compliance with NIH recommendations for the Care and Use of Laboratory Animals; all protocols were approved by the Harvard Medical Area Standing Committee on Animals (Protocol #: 04833). Female *Hhip* ^+/−^ heterozygotes on a C57/BL6 background and their female wild type littermates were subjected to either mixed main-stream and side-stream cigarette smoke (3R4F Kentucky Research cigarettes) for 5 days per week or filtered room air starting at age 10 weeks for a total duration of 6 months.

Plasma, urine, and lung tissue from five mice from each experimental condition (room air-exposed *Hhip* ^+/+^, CS-exposed *Hhip* ^+/+^, room air-exposed *Hhip* ^+/−^, and CS-exposed *Hhip* ^+/−^) were subjected to metabolite profiling. Urine was collected immediately prior to euthanization by placing each mouse on a clean surface and aspirating spontaneously voided urine using a sterile pipette. Urine was transferred into a sterile 1.5 mL centrifugation tube and was immediately stored at −80 °C until analysis. A new, clean surface was used for each mouse. Euthanization by CO_2_ narcosis and cervical dislocation was then performed and 500 μL of blood was collected from the right ventricle immediately using a 26-gauge needle. Blood was transferred into 1.5 mL tubes coated with heparin and centrifuged (2000 × g, 5 minutes, 4 °C) to separate plasma. Plasma aliquots were transferred into new 1.5 tubes and stored immediately at −80 °C until analysis.

Murine lungs were harvested after blood collection for mean alveolar chord length (MACL) measurements as described previously^8^ (also, see below) and were snap-frozen for metabolomic profiling. Lung tissue was homogenized in 6 volumes of water using a bead mill (TissueLyser II; Qiagen Inc.; Valencia, ca) and the aqueous homogenate (30 μL) was subjected to protein precipitation using four volumes of 80% methanol containing inosine-15N4, thymine-d4, and glycocholate-d4 internal standards (Cambridge Isotope Laboratories; Andover, MA). Samples were then centrifuged (9,000 × g, 10 minutes, 4 °C) and aliquots of the supernatant (10 μL each) were subjected to LC-MS profiling methods (below).

### Metabolomic profiling

Untargeted metabolite profiling was performed using liquid chromatography tandem mass spectroscopy (LC/MS-MS) as previously described^36^; additional details are also provided in the Online Supplement – Methods section. MultiQuant software (v1.2) was used for automated peak integration; all peaks were manually reviewed and compound identities were confirmed using reference standards and reference samples^37^. Lung tissue profiles were adjusted for tissue dry weight and urinary profiles were normalized by creatinine levels (assayed as one of the metabolites included in our mass spectroscopy-based platform).

### Data cleaning and analysis

Data cleaning and formatting were performed in MetaboAnalyst 3.0^9^ and R (version 3.2.2, base package). Samples with >40% missingness and metabolites with >30% missingness were removed; remaining missing values were replaced with a small value (one half the minimum detected peak area of the metabolite). Data were log transformed and Pareto scaled prior to conducting univariate analysis by genotype (*Hhip* ^+/+^ versus *Hhip* ^+/−^) or smoke exposure (room air versus CS). Metabolites which demonstrated a minimum fold change of 2 *and* a Students t-test p-value < 0.05 were considered significant. Metabolites that additionally met statistical significance following correction for multiple testing using an false-discovery rate (FDR) < 0.05 were denoted in the results table. Gene-by-smoking interactions and associations between mean alveolar chord length and metabolites were tested using empiric Bayes-mediated linear models as implemented in the *limma* ^38^ package for R. An association p-value < 0.05 was considered significant.

### Metabolite Set Enrichment and Pathway Analysis

Metabolite set enrichment analysis (MSEA) and pathway analyses were performed using MetaboAnalyst 3.0^9^. Over-representation analysis (ORA) was performed using the hypergeometric test using metabolite sets based on normal metabolic pathways and the reference pathway library for *Mus musculus* available through the Kyoto Encyclopedia of Genes and Genomes (KEGG)^39^, respectively. An FDR ≤ 0.05 was considered significant.

### Validation/replication of selected metabolites

Validation of several metabolites was performed on plasma and urine samples collected from a partially overlapping group of mice (n = 20). Cotinine concentrations were determined in the plasma and urine of *Hhip* ^+/+^ and *Hhip* ^+/−^ mice using the Calbiotech Mouse/Rat Cotinine enzyme-linked immunosorbent assays (ELISA) kit (Catalog Number C096D-100, Spring Valley, CA, USA). Quantitative measurements of urine creatine were made using the Abcam colorimetric/fluorometric creatine assay kit (ab65339, Cambridge, MA) in *Hhip* ^+/+^ and *Hhip* ^+/−^ mice exposed to chronic cigarette smoke. Standard curves were generated according to the manufacturer’s guidelines for each assay. Comparisons between groups were made using a Student’s t-test with a p-value < 0.05 to denote significance.

## Electronic supplementary material

Online Supplement
