# Supplementary material for: Metabolomic profiling in a Hedgehog Interacting Protein (Hhip) murine model of chronic obstructive pulmonary disease
Source: Sci Rep. 2017 May 31;7:2504. doi: 10.1038/s41598-017-02701-4 (PMC5451406; doi:10.1038/s41598-017-02701-4)
Supplement: Supplementary file 1 — Online Supplement [file 41598_2017_2701_MOESM1_ESM.pdf]

Metabolomic profiling in a Hedgehog Interacting Protein (Hhip) murine model of chronic obstructive pulmonary disease.

Emily S. Wan, Yan Li, TaoTao Lao, Weiliang Qiu, Zhiqiang Jiang, John D. Mancini, Caroline A. Owen, Clary Clish, Dawn L. DeMeo, Edwin K. Silverman, Xiaobo Zhou

Online Data Supplement

Supplementary Methods

Metabolite Profiling

Three LC-MS methods were used to measure polar metabolites and lipids in plasma, urine, and lung tissue homogenates from Hhip heterozygote and wild type mice. Lung tissue samples were homogenized in 6 volumes of water using a bead mill (TissueLyser II; Qiagen Inc.; Valencia CA) and the aqueous homogenate was aliquoted for each method. Negative ion mode, targeted MS analyses of polar metabolites were conducted as described previously[1]. Briefly, LC-MS samples were prepared from body fluids or tissue homogenates (30 µL) via protein precipitation with the addition of four volumes of 80% methanol containing inosine-15N4, thymine-d4 and glycocholate-d4 internal standards (Cambridge Isotope Laboratories; Andover, MA). The samples were centrifuged (10 min, 9,000 x g, 4°C) and the supernatants were analyzed using an ACQUITY UPLC (Waters, Milford MA) coupled to a 5500 QTRAP triple quadrupole mass spectrometer (AB SCIEX, Framingham, MA). Extracts (10 µL) were injected directly onto a 150 x 2.0 mm Luna NH2 column (Phenomenex; Torrance, CA). The column was eluted at a flow rate of 400 µL/min with initial conditions of 10% mobile phase A (20 mM ammonium acetate and 20 mM ammonium hydroxide in water) and 90% mobile phase B (10 mM ammonium hydroxide in 75:25 v/v acetonitrile/methanol) followed by a 10 min linear gradient to 100% mobile phase. MS data were acquired using multiple reaction monitoring scans tuned for each compound using authentic reference standards. The ion spray voltage was -4.5 kV and the source temperature was 500°C. Raw data were processed using MultiQuant 2.1 software (SCIEX, Framingham MA). Nontargeted, positive ion mode analyses of polar metabolites and lipids were conducted using two separate methods as described previously[2]. Data for both methods were acquired using a Nexera X2 U-HPLC system (Shimadzu Scientific Instruments; Marlborough, MA) coupled to a Q Exactive orbitrap mass spectrometer (Thermo Fisher Scientific; Waltham, MA). Polar metabolites were extracted from body fluids and tissue homogenates (10 µL) using addition of nine volumes of 74.9:24.9:0.2 v/v/v acetonitrile/methanol/formic acid containing stable isotope-labeled internal standards (valine-d8, Isotec; and phenylalanine-d8, Cambridge Isotope Laboratories; Andover, MA). The extracts were centrifuged (10 min, 9,000 x g, 4°C), and the supernatants were injected onto a 150 x 2 mm Atlantis HILIC column (Waters; Milford, MA). The column was eluted isocratically at a flow rate of 250 µL/min with 5% mobile phase A (10 mM ammonium

formate and 0.1% formic acid in water) for 1 minute followed by a linear gradient to 40% mobile phase B (acetonitrile with 0.1% formic acid) over 10 minutes. Polar metabolite MS analyses were carried out using electrospray ionization in the positive ion mode using full scan analysis over m/z 70-800 at 70,000 resolution and 3 Hz data acquisition rate. Additional MS settings were: ion spray voltage, 3.5 kV; capillary temperature, 350°C; probe heater temperature, 300 °C; sheath gas, 40; auxiliary gas, 15; and S-lens RF level 40. Lipids were extracted from plasma and lung tissue homogenates (10 µL) using 190 µL of isopropanol containing 1-dodecanoyl-2-tridecanoyl-sn-glycero-3-phosphocholine as an internal standard (Avanti Polar Lipids; Alabaster, AL). After centrifugation (10 min, 9,000 x g, ambient temperature), supernatants (2 µL) were injected directly onto a 100 x 2.1 mm ACQUITY BEH C8 column (1.7 µm; Waters; Milford, MA). The column was eluted at a flow rate of 450 µL/min isocratically for 1 minute at 80% mobile phase A (95:5:0.1 vol/vol/vol 10 mM ammonium acetate/methanol/acetic acid), followed by a linear gradient to 80% mobile-phase B (99.9:0.1 vol/vol methanol/acetic acid) over 2 minutes, a linear gradient to 100% mobile phase B over 7 minutes, and then 3 minutes at 100% mobile-phase B. MS analyses were carried out using electrospray ionization in the positive ion mode using full scan analysis over m/z 200-1100 at 70,000 resolution and 3 Hz data acquisition rate. Additional MS settings were: ion spray voltage, 3.0 kV; capillary temperature, 300°C; probe heater temperature, 300 °C; sheath gas, 50; auxiliary gas, 15; and S-lens RF level 60. Raw data from the three nontargeted methods were processed using Progenesis QI software (NonLinear Dynamics) for feature alignment, nontargeted signal detection, and signal integration. Targeted processing of a subset of known metabolites was conducted using TraceFinder 3.0 software (Thermo Fisher Scientific; Waltham, MA). Compound identities were confirmed using reference standards and reference samples[3].

Supplementary Table S1 – Metabolites with differential concentrations by Hhip genotype (Hhip$^{+/-}$ versus Hhip$^{+/+}$) in room air-exposed mice.

**Biomaterial / Metabolite**	**Log2 (Fold change)***	**P-value†**
Plasma		
C6 carnitine	-2.58	0.02
C8 carnitine	-1.41	0.02
C12 carnitine	-0.64	0.04
C58:12 TAG	0.83	0.04
quinolinate	-0.68	0.02
Urine		
2-deoxyadenosine	-1.17	0.04
Adenine	1.50	0.01
alpha-glycerophosphate	-0.80	0.02
alpha-glycerophosphocholine	-0.72	0.01
aminoisobutyric acid	-0.69	0.03
butyrobetaine	-0.91	0.01
cAMP	-0.78	0.01
glycocholate-d4	0.90	0.01
Histidine	1.14	0.01
hydroxyphenylpyruvate	-0.64	0.02
inosine-15N4	0.91	0.01
isoleucine	-0.64	0.02
oxalate	0.85	0.04
phenylalanine-d8	0.74	1.19×10^{-3}
pyroglutamic acid	-1.30	0.01
valine-d8	0.72	2.96×10^{-3}
**Lung**		
fructose/glucose/galactose	0.67	0.02
gentistate	0.75	3.18×10^{-3}
lactose	0.72	0.01
sucrose	0.64	0.01

AMP = adenosine monophosphate, TAG = triacylglyceride.

* Negative values indicate *lower* concentration in Hhip$^{+/-}$ heterozygotes (minimum 1.5x fold change)

† Student's t-test

Supplementary Table S2 – Metabolites with differential concentrations following exposure to chronic cigarette smoke exposure in wild-type ($Hhip^{+/+}$) mice

**Biomaterial / Metabolite**	**Log2 (Fold change)***	**P-value†**
Plasma		
Cotinine	5.20	2.48×10^{-3}
Glutathione (oxidized)	-1.01	0.03
quinolinate	-0.80	0.03
thiamine	-0.67	0.04
uridine	-0.67	0.03
Urine		
1-methylhistamine	-1.07	2.91×10^{-3}
1-methylnicotinamide	0.77	0.01
4-hydroxybenzaldehyde	-0.79	0.05
5-aminolevulinic acid	2.42	0.02
Adenine	1.46	7.34×10^{-3}
Allantoin	-0.85	7.97×10^{-3}
Cotinine	4.61	7.69×10^{-4} ɫ
Creatine	-2.54	7.52×10^{-4} ɫ
erythrose-4-phosphate	0.81	0.04
glucuronate	0.81	0.03
glycocholate-d4	0.78	0.02
Guanine	1.53	2.38×10^{-6} ɫ
inosine-15N4	0.78	0.05
N-carbamoyl-beta-alanine	-1.04	0.02
Nicotinate	4.36	0.02
Oxalate	1.17	0.05
Pantothenate	-1.78	0.04
suberate	-0.77	0.05
valine-d8	0.68	0.04
Xanthine	1.34	6.16×10^{-3}
xanthosine	-0.65	0.02
**Lung**		
C30:1 PC	0.72	0.04
C32:2 PC	0.64	0.05

PC = phosphatidylcholine

* Negative values indicate *lower* concentration in wild type $Hhip^{+/+}$ mice exposed to chronic cigarette smoke (minimum 1.5x fold change)

† Student's t-test p-value

ɫ Denotes significance at a false discovery rate (FDR) < 0.05

Supplementary Table S3 – Metabolites with differential concentrations following exposure to chronic cigarette smoke exposure in ($Hhip^{+/-}$) heterozygote mice

**Biomaterial / Metabolite**	**Log2 (Fold change)***	**P-value†**
Plasma		
4-hydroxybenzaldehyde	1.57	0.03
AMP	2.33	0.02
Cotinine	3.95	1.55×10^{-3}
Cytidine	1.39	0.02
Gentisate	-1.44	0.01
Glucose	-1.06	0.04
Glutamate	1.29	0.01
GMP	1.85	0.02
Threonine	1.11	0.04
UMP	2.53	0.03
Urine		
3-hydroxybenzoate	-0.69	0.05
ADP	1.13	0.03
Alpha-ketoglutarate	-1.55	0.02
AMP	1.52	0.02
Argininosuccinate	-1.72	1.05×10^{-3}
C3 carnitine	-0.90	0.04
C3-DC-CH3 carnitine	1.38	0.04
cAMP	0.65	0.02
Carnosine	-1.73	0.04
choline	-0.90	0.02
Cotinine	2.56	0.02
fumarate/maleate/alpha-ketoisovalerat	-0.93	0.03
Glutamate	1.02	0.03
Guanine	1.13	9.67×10^{-3}
Histidine	-1.34	3.95×10^{-3}
hydroxyphenylacetate	-0.71	0.04
inositol	0.69	6.27×10^{-3}
Lactate	-1.22	0.01
Malate	-1.24	0.03
NMMA	-0.78	0.05
Pantothenate	-1.41	0.02
Putrescine	-1.75	0.03
Succinate	-1.05	5.83×10^{-3}
tyrosine	-0.60	0.04
XMP	1.91	0.02
Lung		
Adenylosuccinate	1.41	4.98×10^{-3}
C26 carnitine	0.60	0.04
gentistate	-0.65	0.03
lactose	-0.69	0.04
malondialdehyde	-0.94	0.02
sucrose	-0.64	0.05

ADP = adenosine diphosphate, AMP = adenosine monophosphate, NMMA = N-monomethylarginine

* Negative values indicate *lower* concentration in $Hhip^{+/-}$ mice exposed to chronic cigarette smoke (minimum 1.5x fold change)

† Student's t-test

Supplementary Table S4 – Metabolites with differential concentrations by Hhip genotype (Hhip$^{+/-}$ versus Hhip$^{+/+}$) in cigarette smoke-exposed mice.

**Biomaterial / Metabolite**	**Log2 (Fold change)***	**P-value†**
Plasma		
benzoate	0.99	0.03
C30:1 phosphatidylcholine	-1.51	0.04
C46:1 TAG	-0.90	0.04
C48:1 TAG	-0.63	0.01
C48:2 TAG	-0.64	0.02
C48:3 TAG	-0.72	0.04
C50:0 TAG	-0.70	0.04
C50:6 TAG	-0.60	0.04
C52:0 TAG	-0.68	0.03
C52:7 TAG	-0.61	0.05
C54:1 TAG	-0.61	0.04
inositol	0.67	0.04
Pantothenate	1.16	3.6×10^{-3}
Sorbitol	1.39	0.05
thymine	0.71	0.02
Urine		
Alpha-hydroxybutyrate	-1.22	0.04
C5 carnitine	-1.82	0.03
Cotinine	-1.89	0.03
Creatine	1.17	0.03
Lung		
2-aminoadipate	-0.63	0.03
butyrobetaine	-0.65	0.02
C30:1 PC	-0.75	0.02
C32:2 PC	-0.77	0.01
cytosine	-0.94	0.02
hydroxyphenylacetate	-0.60	0.02
lactose	-0.60	0.04

TAG = triacylglycerol, PC = phosphatidylcholine

* Negative values indicate *lower* concentration in Hhip$^{+/-}$ heterozygotes (minimum 1.5x fold change)

† Student's t-test

Supplementary Table S5 – Metabolites in plasma associated ($p<0.05$) with lung mean alveolar chord length (MACL) measurements.

	Room Air		Chronic cigarette smoke	
$Hhip^{+/+}$ - Wild type	N = 5	Direction of effect	N = 5	Direction of effect
	C56:10 TAG	+	C56:3 TAG	+
	C58:10 TAG	+	C18:3 CE	+
	C56:8 TAG	+	C56:4 TAG	+
	C24:1 SM	+	C54:2 TAG	+
	C20:5 CE	-	C56:2 TAG	+
	C56:9 TAG	+	beta-hydroxybutyrate	-
	C20:3 CE	-	C18:2 LPC	-
	trimethylamine-n-oxide	+	C32:0 PC	-
	C56:7 TAG	+	C4 carnitine	+
	C58:9 TAG	+	C30:0 PC	-
	C54:9 TAG	+		
$Hhip^{+/-}$ Heterozygote	N = 5	Direction of effect	N = 5	Direction of effect
	taurine	+	C16:0 CE	-
	C60:12 TAG	-	4-pyridoxate	+
			C16:0 LPC	-
			Fructose-1-phosphate/fructose-6-phosphate/glucose-1-phosphate/glucose-6-phosphate	-
			C20:3 LPC	-

(+) denotes a positive correlation between metabolite concentration and MACL measurements (i.e. increasing severity of emphysema associated with higher metabolite level). TAG = triacylglyceride, CE = cholesterol ester, SM = sphingomyelin, LPC = lysophosphorylcholine, PC = phosphatidylcholine.

Supplementary Table S6 – Metabolites in urine associated ($p<0.05$) with lung mean alveolar chord length (MACL) measurements.

	Room Air		Chronic cigarette smoke	
Hhip $^{+/+}$ - Wild type	N = 4	Direction of effect	N = 5	Direction of effect
	Alpha-glycerophosphocholine	-	nicotinate	-
	lactose	+	glycine	-
	pyroglutamic acid	-		
	phosphoenolpyruvate	-		
	allantoin	-		
	phosphocholine	-		
	alpha-glycerophosphate	-		
	xanthosine	-		
	creatine	-		
Hhip $^{+/-}$ Heterozygote	N = 5	Direction of effect	N = 4	Direction of effect
	adenosine	+	thiamine	+
	hydroxyphenylpyruvate	+	methionine sulfoxide	+
	histidine	-	3-hydroxybenzoate	+
	fructose-1-phosphate/fructose-6-phosphate/glucose-1-phosphate/glucose-6-phosphate	+	lactose	+
			trimethylamine-N-oxide	+
			carnitine	+
	arginine	-	methylnicotinamide	-
	carnitine	-	guanosine	-
	guanosine	+	C2 carnitine	+

(+) denotes a positive correlation between metabolite concentration and MACL measurements (i.e. increasing severity of emphysema associated with higher metabolite level).

Supplementary Table S7 – Metabolites in lung associated ($p<0.05$) with lung mean alveolar chord length (MACL) measurements.

	Room Air				Chronic cigarette smoke	
Hhip $^{+/+}$ - Wild type	N = 4	Direction of effect		Direction of effect	N = 5	Direction of effect
	AMP	+	C54:3 TAG	-	C18:1 carnitine	+
	C50:1 TAG	-	Fructose/glucose/galactose	-	C18:2 carnitine	+
	C52:2 TAG	-	UMP	+	orotate	-
	C36:2 DAG	-	C54:1 TAG	-	anthranilic acid	-
	C18:2 LPC	-	C20:5 CE	+		
	C48:0 TAG	+	C20:5 LPC	+		
	carnitine	-	C46:2 TAG	-		
	C52:1 TAG	-	glutamine	+		
	C34:1 DAG	-	C36:1 DAG	-		
	taurine	-	Fumarate/maleate/α-ketoisovalerate	-		
	C56:1 TAG	-				
	C22:6 CE	+	C44:0 TAG	-		
	C54:2 TAG	-	C46:1 TAG	-		
	C34:4 PC	-	C20:3 CE	+		
	C48:1 TAG	-	C48:3 TAG	-		
	C52:3 TAG	-	C56:3 TAG	-		
	C50:3 TAG	-	creatine	-		
	C56:5 TAG	+	C58:6 TAG	+		
	C50:0 TAG	-	C34:0 PC	+		
	C20:4 CE	+	C52:0 TAG	-		
	C46:0 TAG	-	C32:2 DAG	-		
	C48:2 TAG	-	C44:1 TAG	-		
	C18:2 CE	+	C50:4 TAG	-		
	C56:2 TAG	-	GMP	+		
	C36:3 DAG	-	C44:2 TAG	-		
Hhip $^{+/-}$ Heterozygote	N = 4			Direction of effect	N = 5	Direction of effect
	phosphoglycerate			-	C40:9 PC	-
	C18 carnitine			-	C38:6 PC	-
	α-glycerophosphocholine			-	C22:6 LPC	-
	creatinine			-	C32:2 PC	-
	C20:4 LPE			+	C34:2 DAG	-
	glutamine			-	C16:0 LPC	-
	C18:1 carnitine			-	C18:2 LPC	-
	5-adenosylhomocysteine			-	C34:2 PC	-
					phosphoenolpyruvate	-
					malondialdehyde	-

			C20:4 LPE<br>C18:0 LPC<br>sorbitol	-<br>-<br>-

(+) denotes a positive correlation between metabolite concentration and MACL measurements (i.e. increasing severity of emphysema associated with higher metabolite level). TAG = triacylglyceride, CE = cholesterol ester, SM = sphingomyelin, LPC = lysophosphorylcholine, PC = phosphatidylcholine, LPE = lysophosphatidylethanolamine .

Supplementary Figure S1 – Venn diagram illustrating the overlap between metabolites detected in plasma (red), urine (yellow), and lung (blue) from all mice.

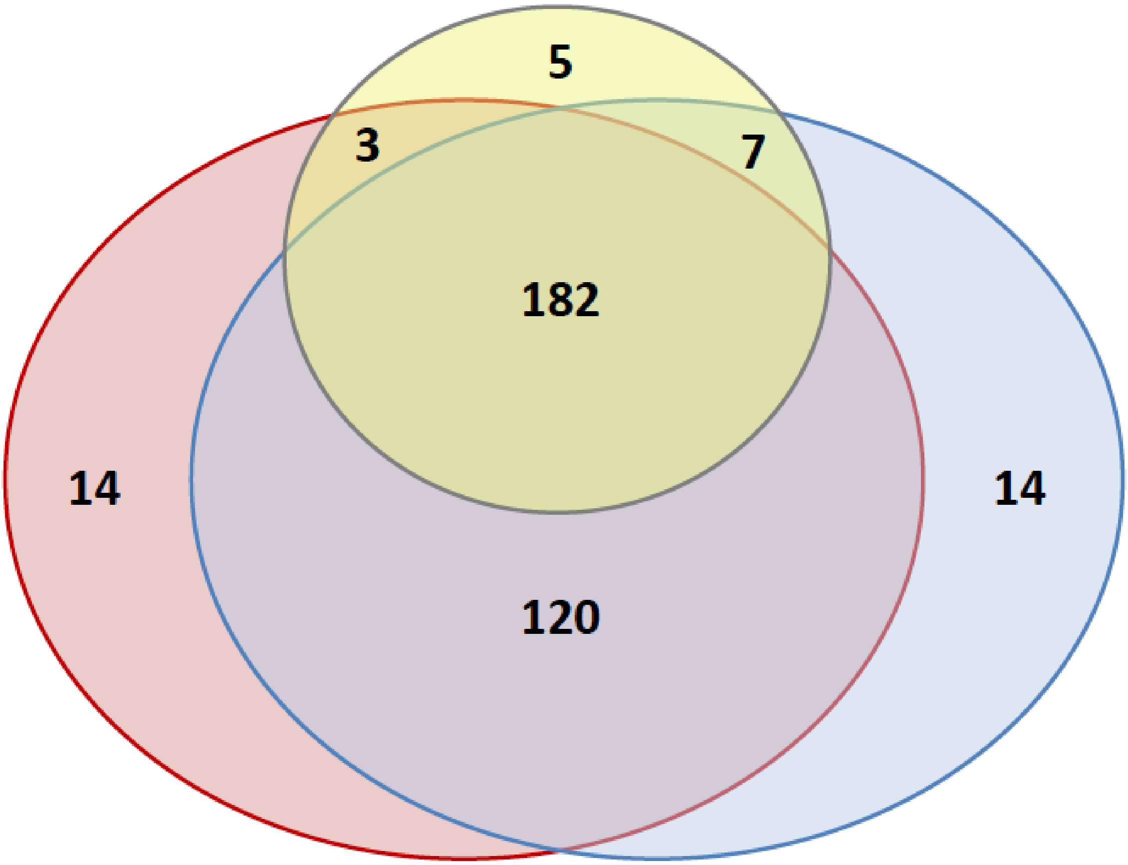

Supplementary Figure S2 - Pathway analysis of overlapping metabolites (4) identified in gene-by-environment analyses in plasma and urine. The results of pathway analysis with pathway impact on the x-axis and –log(p-value) plotted on the y-axis are illustrated in panel (a). Panels (b) and (c) show the KEGG glyoxalate and dicarboxylate and citrate cycle pathways, respectively. The location of metabolites identified in our analyses are highlighted in red.

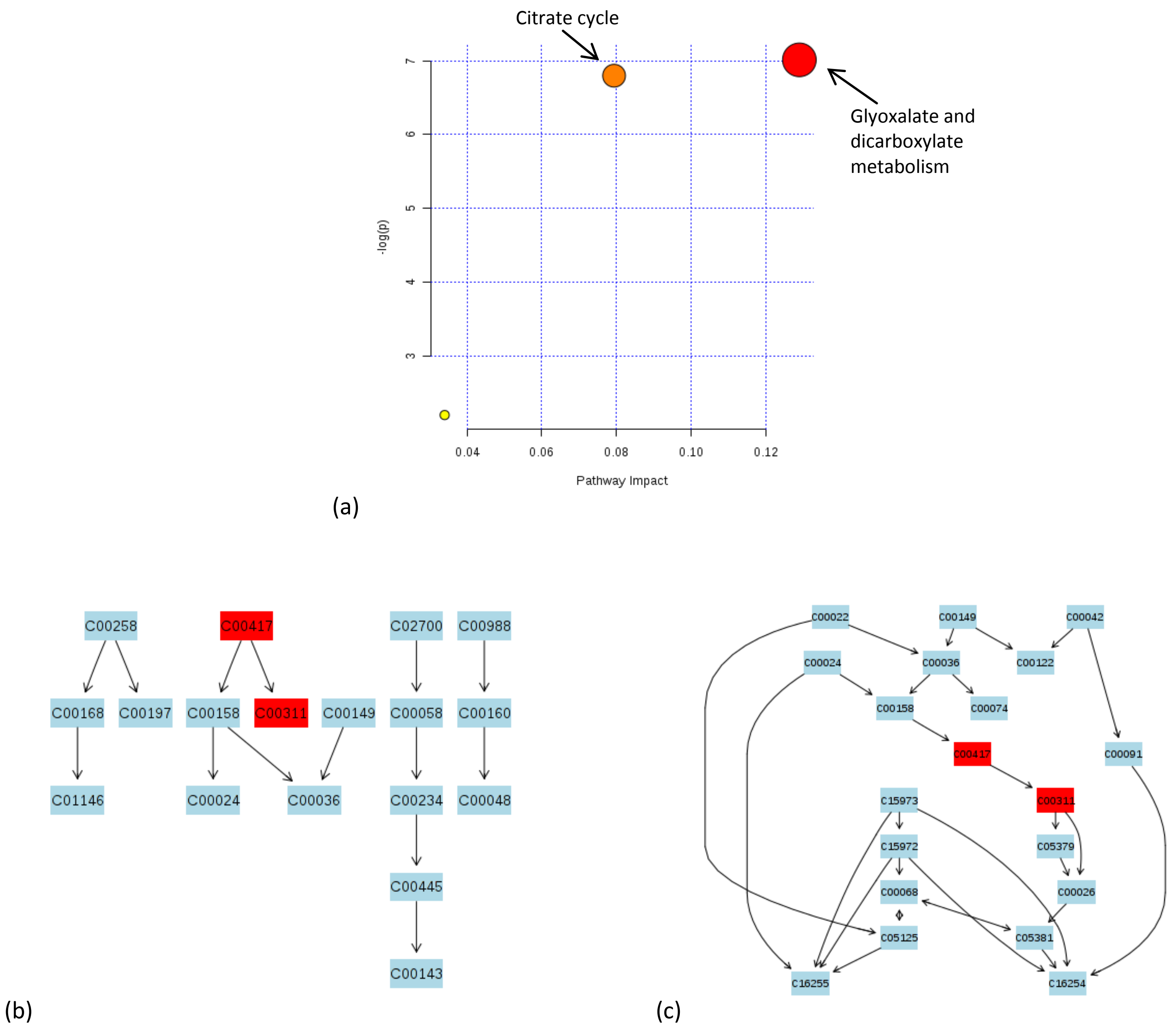

Supplementary Figure S3 – Quantitative urinary cotinine levels as assessed using ELISA. Significantly increased concentrations of cotinine were observed in *Hhip*$^{+/+}$ (n = 5) and *Hhip*$^{+/-}$ (n = 5) mice exposed to chronic cigarette smoke relative to *Hhip*$^{+/+}$ (n = 5) and *Hhip*$^{+/-}$ (n = 5) mice exposed to room air, respectively. No difference was observed by genotype between Hhip$^{+/+}$ and HHip$^{+/-}$ mice exposed to chronic cigarette smoke.

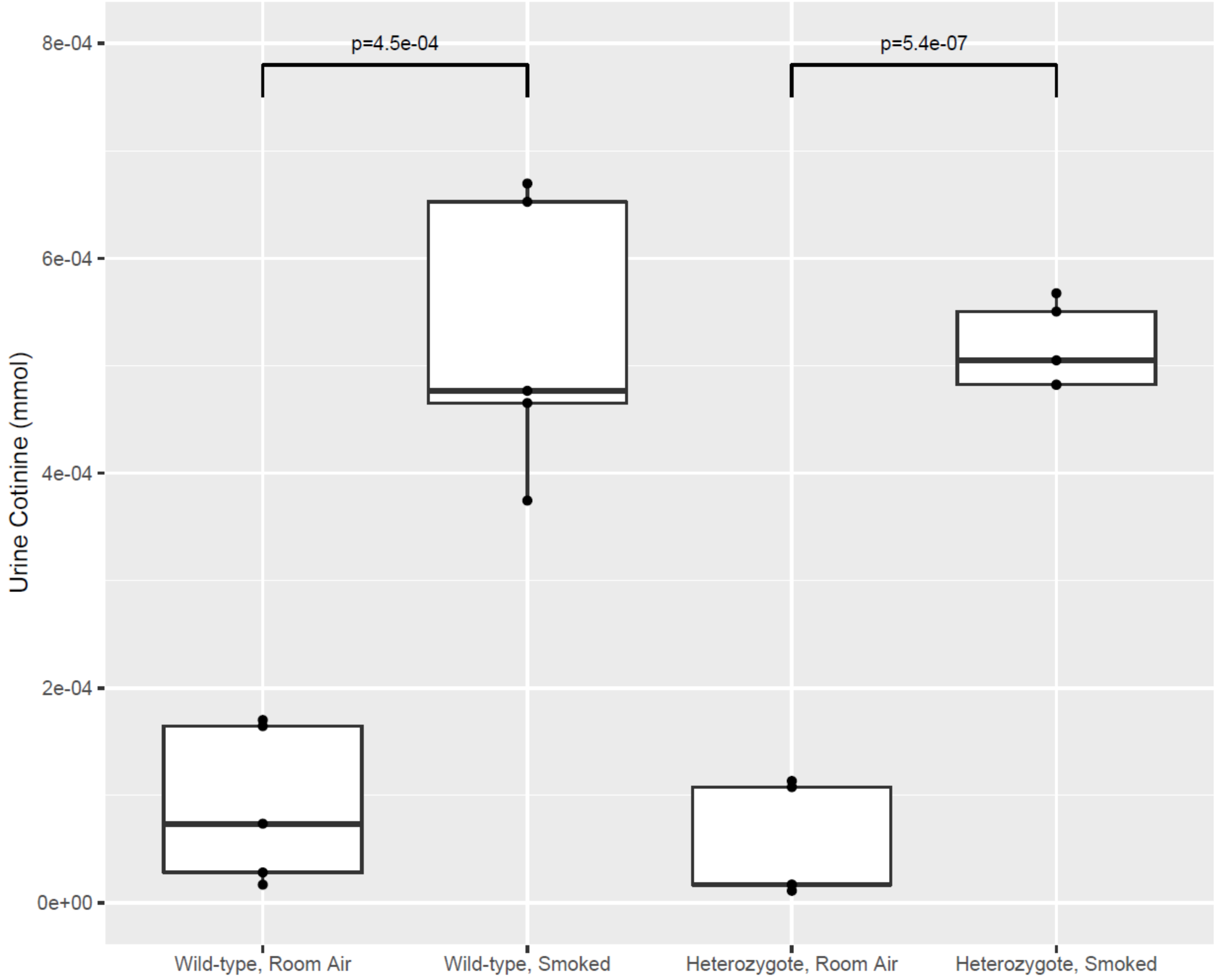

Supplementary Figure S4– Quantitative plasma cotinine levels as assessed using ELISA. A trend towards increased serum cotinine was observed in *Hhip*$^{+/+}$ (n = 5) and *Hhip*$^{+/-}$ (n = 5) mice exposed to chronic cigarette smoke relative to *Hhip*$^{+/+}$ (n = 5) and *Hhip*$^{+/-}$ (n = 5) mice exposed to room air, respectively.

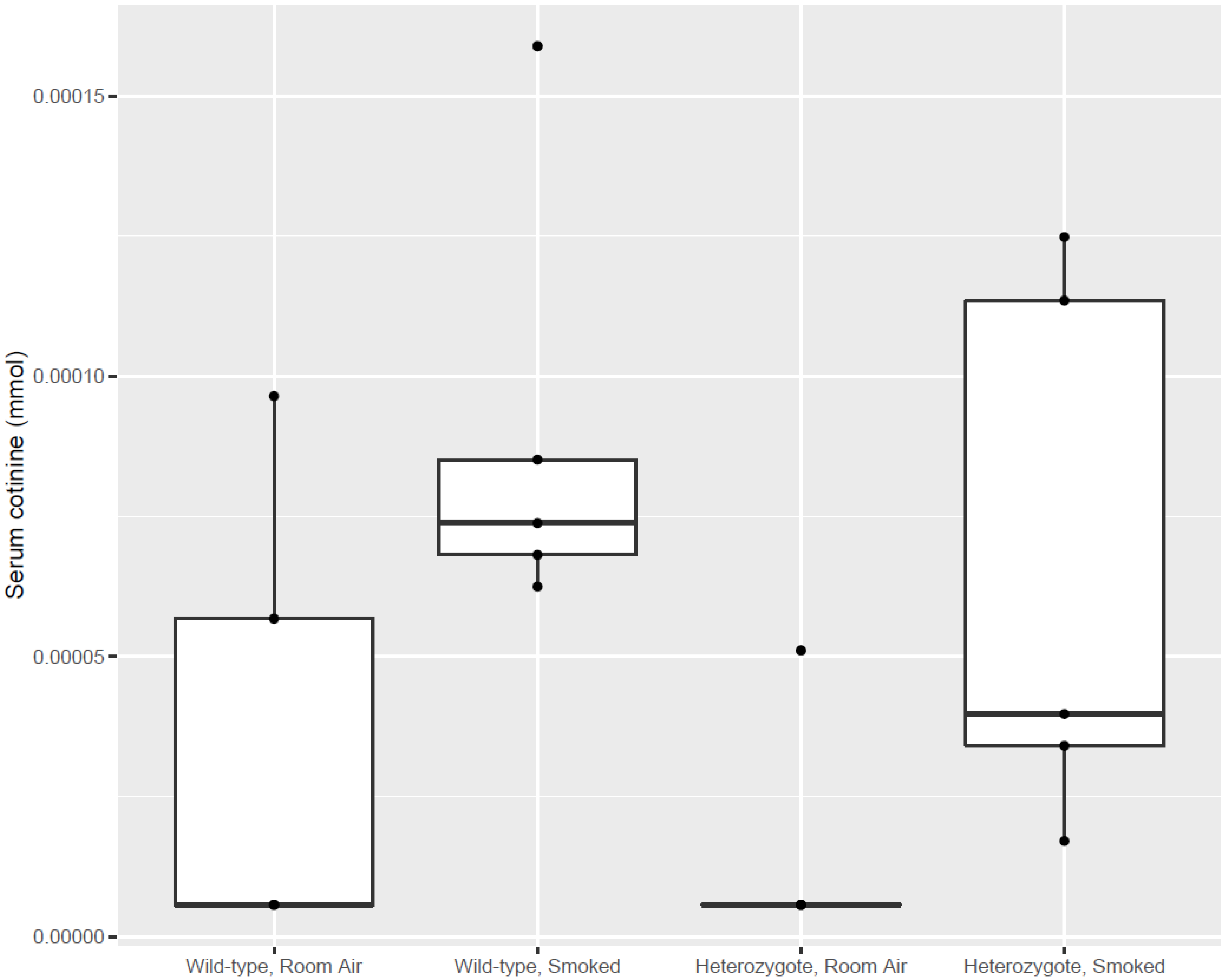

Supplementary Figure S5 – Quantitative urinary creatine levels as assessed using colorimetric assays. Exposure to chronic cigarette smoke caused a significant decrease in urinary creatine excretion in *Hhip*$^{+/+}$ mice (n = 5), but not in *Hhip*$^{+/-}$ mice (n = 5). No differences in urinary creatine excretion by genotype were noted in *Hhip*$^{+/+}$ (n = 5) and *Hhip*$^{+/-}$ (n = 5) mice exposed to chronic cigarette smoke.

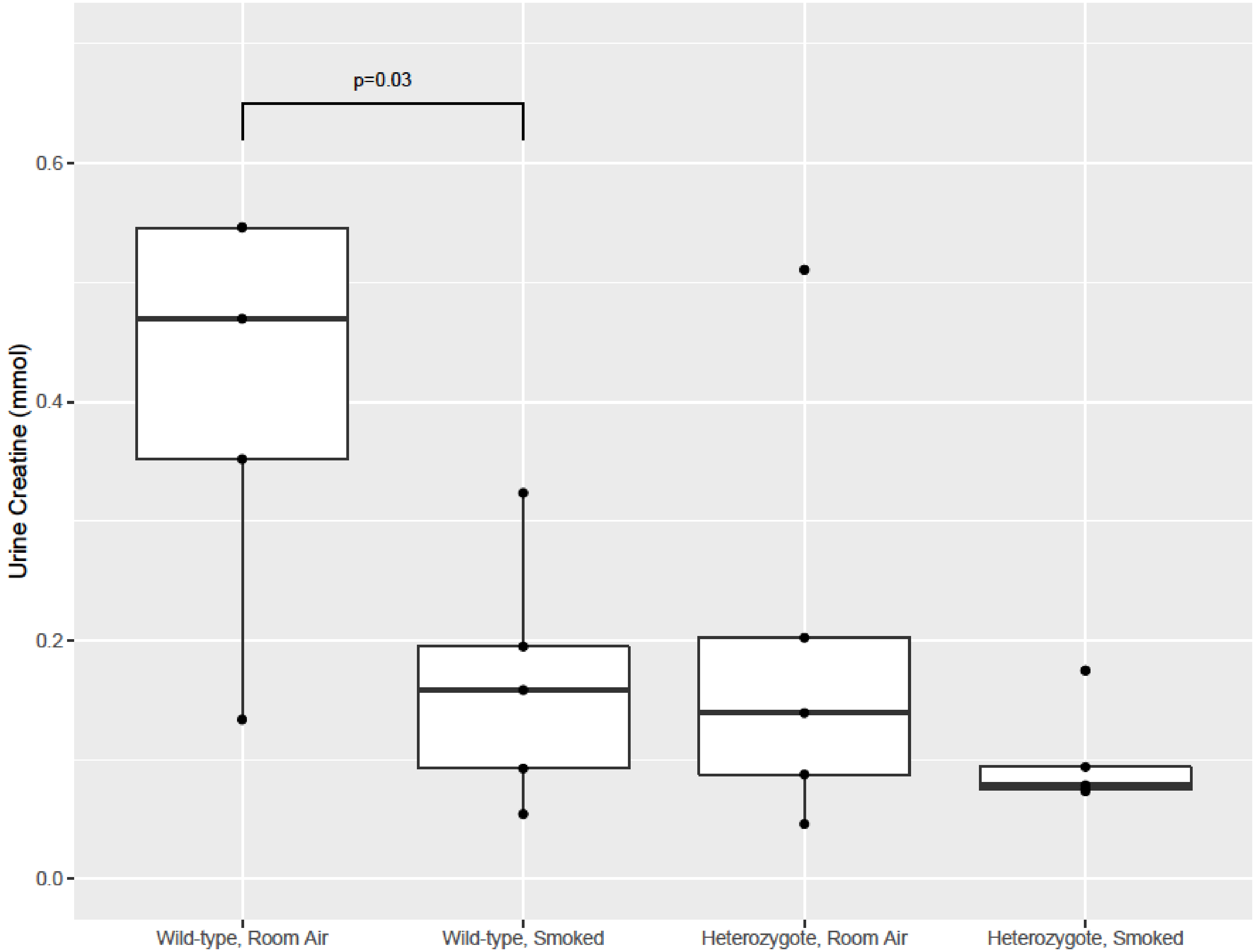

Supplementary References

1 Townsend, M. K. *et al.* Reproducibility of metabolomic profiles among men and women in 2 large cohort studies. *Clin Chem* **59**, 1657-1667, doi:10.1373/clinchem.2012.199133 (2013).

2 Mascanfroni, I. D. *et al.* Metabolic control of type 1 regulatory T cell differentiation by AHR and HIF1-alpha. *Nat Med* **21**, 638-646, doi:10.1038/nm.3868 (2015).

3 Sumner, L. W. *et al.* Proposed minimum reporting standards for chemical analysis Chemical Analysis Working Group (CAWG) Metabolomics Standards Initiative (MSI). *Metabolomics* **3**, 211-221, doi:10.1007/s11306-007-0082-2 (2007).
